# The Human Cytomegalovirus, from Oncomodulation to Oncogenesis

**DOI:** 10.3390/v10080408

**Published:** 2018-08-03

**Authors:** Georges Herbein

**Affiliations:** 1 Department Pathogens & Inflammation-EPILAB, UPRES EA4266, University of Bourgogne France-Comté (UBFC), F-25030 Besancon, France; georges.herbein@univ-fcomte.fr; Tel.: +33-381-665-552; 2 Department of Virology, CHRU Besancon, F-25030 Besancon, France

**Keywords:** HCMV, cancer, CTH cells, oncomodulation, oncovirus

## Abstract

Besides its well-described impact in immunosuppressed patients, the role of human cytomegalovirus (HCMV) in the pathogenesis of cancer has been more recently investigated. In cancer, HCMV could favor the progression and the spread of the tumor, a paradigm named oncomodulation. Although oncomodulation could account for part of the protumoral effect of HCMV, it might not explain the whole impact of HCMV infection on the tumor and the tumoral microenvironment. On the contrary cases have been reported where HCMV infection slows down the progression and the spread of the tumor. In addition, HCMV proteins have oncogenic properties per se, HCMV activates pro-oncogenic pathways in infected cells, and recently the direct transformation of cells following HCMV infection has been described, which gave rise to tumors when injected in mice. Thus, beyond the oncomodulation model, this review will assess the direct transforming role of HMCV-infected cells and the potential classification of HCMV as an oncovirus.

## 1. Introduction

The human cytomegalovirus belongs to the Herpesviridae family with a double stranded DNA genome of 236 kbp in size [1]. In contrast to previous predictions [2,3], the translated products from open reading frames (ORF) in human cytomegalovirus (HCMV) genome are much more numerous than previously believed because of the presence of viral short ORFs, alternative splicing, and translation on cytosolic transcripts outside of conserved reading frames [4]. Several cellular functions involved in tumor development are targeted by HCMV gene products including cell cycle dysregulation, cellular immortalization, mutation and instability of the viral genome, enhanced cell survival, and immune escape with tumor spread [5,6,7,8]. In addition, HCMV infects several cell types present in tumoral tissue and microenvironment.

Most organs and tissues of the human body can be infected by HCMV. Although the replication of highly passaged laboratory HCMV strains is limited to fibroblasts, HCMV low passage clinical isolates exhibit an extended cellular tropism for epithelial cells, endothelial cells, hepatocytes, fibroblasts, stromal cells, monocytes/macrophages, astrocytes, and neural stem/progenitor cells [9,10,11,12,13]. Epithelial cells present in lung, breast, gastrointestinal tract, and kidney can be targeted by HCMV. HCMV infects human lung epithelial cells in vitro with release of newly produced virions up to eight weeks post-infection with a typical cytopathic effect [14]. Human mammary epithelial cells (HMECs) are productively infected by HCMV clinical isolates with low levels of replication [15,16]. HCMV also replicates in renal epithelial cells [16] and hepatocytes are permissive for HCMV replication with a limited viral production [12,17,18]. After prolonged infection in vitro of human embryonic fibroblasts, large syncytia appear in cultures with typical HCMV intranuclear inclusion bodies [19]. In fact, low passage clinical HCMV strains have an intact ULb’ sequence, the region at the right end of the Unique Long region (UL) genome component, which is absent in laboratory adapted HCMV strains. The ULb’ sequence is critical for the viral tropism and favors the replication of HCMV in several primary cell types including epithelial cells, endothelial cells, and myeloid cells [13,20]. On the contrary, laboratory adapted HCMV strains such as AD169 have lost fully or partially the ULb’ region and have a restricted tropism for fibroblasts. Besides epithelial cells and fibroblasts, HCMV infects persistently monocytes/macrophages, which behave like a viral reservoir and favor the viral spread through the body [21,22]. Upon HCMV infection of monocytes, activation of NF–kB and PI3K pathways results in a M1/M2 phenotype with both inflammatory and immunosuppressive profiles [21]. Inflammatory factors including Tumor Necrosis Factor (TNF)-alpha, interleukin-6 (IL-6), and nitric oxide synthase 2 are produced by M1 macrophages following HCMV infection [21]. Similarly, an enhanced secretion of TNF-alpha, IL-6, and chemokines is detected in supernatants from CMV-stimulated purified microglial cell cultures [23]. Increased production of proinflammatory cytokines could favor the development of cancer (reviewed in the work of [24]). Infection of astrocytes with CMV results in the enhanced production of chemokines MCP-1 and IL-8, which attract macrophages/microglia in their vicinity [23]. CMV infection of astrocytes turns on TGF-beta production, which exerts positive feedback on viral replication [25]. Altogether, HCMV infects epithelial cells, myeloid cells, fibroblasts, and central nervous system (CNS) cells, all of which could participate to the tumor formation and the tumoral microenvironment.

HCMV may enhance the malignancy of cancer cells and/or tumor-associated cells, a paradigm named oncomodulation [26,27,28]. Although HCMV-induced oncomodulation has been extensively studied so far, the direct involvement of HCMV in cell transformation and identifying viral genes favoring such a transformation could define HCMV as an oncovirus.

## 2. Oncomodulation by HCMV

### 2.1. The Paradigm of Oncomodulation

On the one hand, the hypothesis of HCMV-induced oncomodulation is supported by the detection of viral proteins and DNA in cancer tissues including glioma, colorectal cancer, prostate cancer, breast cancer, mucoepidermoid carcinoma, medulloblastoma, and neuroblastoma [29,30,31,32,33,34,35]. On the other hand, neither HCMV antigens nor HCMV genome were detected in high fractions of tumors [36,37,38,39,40,41,42,43]. This apparent discrepancy could result from distinct sensitivity of biological assays used to detect HCMV in the tumor samples. Also, negative results could be due to the fact that the tumor harbors only part of the HCMV genome (similar to the detection of E6 and E7 HPV for cervical cancer), which is not targeted by the conventional HCMV assays that recognize well-known viral gene products (pUL123 (IE1), pUL122 (IE2), pUL83 (pp65)). On the contrary, positive results could result from the detection of HCMV in tumoral tissues like an “opportunistic infection” in already immunosuppressed cancerous patients and may have no direct link with tumor appearance and/or progression.

### 2.2. In Favor of Oncomodulation, HCMV Infection of Established Cancer Cells Favors Malignancy

**Figure 1:**
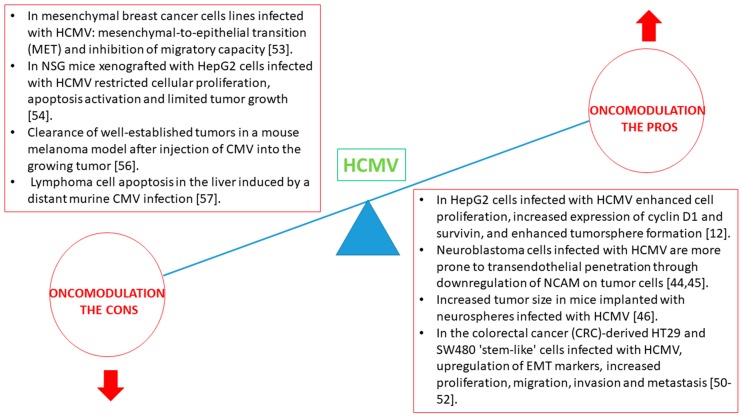
The pros and cons of oncomodulation following human cytomegalovirus (HCMV) infection. NSG—NOD/SCID Gamma; NCAM-Neural Cell Adhesion Molecule.

As oncomodulation is defined as enhanced malignancy following viral infection, it is critical to show that HCMV infection of already transformed cells favors the development of oncogenesis and/or activates pathways implicated in transformation and/or oncogenesis (Figure 1). Thus, the HepG2 human liver cancer cell line with an epithelial morphology isolated from a patient with a hepatocellular carcinoma can be infected with HCMV. Secretion of IL-6 with autocrine/paracrine activation of the IL–6R–JAK–STAT3 pathway is observed in HCMV-infected HepG2 cells. Enhanced cell proliferation occurs in HCMV-infected HepG2 cells parallel to enhanced production of cyclin D1 and survivin [12]. Enhanced production of tumorspheres is observed following HCMV infection of HepG2 cells compared with uninfected cultures [12]. Altogether, HCMV infection of HepG2 liver cells enhances malignant properties of this established liver cancer cell line.

Neuroblastoma cell infected with HCMV are more prone to transendothelial penetration through downregulation of the Neural Cell Adhesion Molecule (NCAM) on tumor cells compared with uninfected cells [44,45]. HCMV infection fuels the tumor formation in mice implanted with neurospheres compared with uninfected neurospheres [46]. Interestingly, proliferation of patient-derived glioblastoma neurospheres was increased by HCMV [46]. In addition, in patient-derived glioma stem-like cells (GSC) infected with HCMV the stemness properties were enhanced because of the expression of IE viral proteins [47]. CMV infection of developing murine brain perturbates the mobility of virus-infected neuronal cells [48,49].

Following HCMV infection of the colorectal cancer (CRC) HT29 and SW480 ‘stem-like’ cells, both EMT and WNT pathways are activated resulting in enhanced cellular proliferation and mobility [50]. In human CRC surgical specimen snail, EMT and CSC-like phenotype are linked to tumor spread [51]. In addition, EMT and CSC-like phenotypes are observed in human CRC cells when twist is overexpressed resulting in increased invasion and tumorsphere formation abilities [52]. We observed EMT features with enhanced snail and twist expression in CMV-transformed human mammary epithelial cells (CTH cells) [15].

### 2.3. Against Oncomodulation, HCMV Infection of Established Cancer Cells Counteracts Malignancy

Besides a positive role for HCMV infection toward enhancement of malignancy, recent reports indicate that the virus can repress the transformation process in cancer cells (Figure 1). In the mesenchymal breast cancer lines MDA-MB-231 and SUM1315, HCMV induces a mesenchymal-to-epithelial transition (MET) with inhibition of their migratory capacity [53]. In addition, in the HCMV infected MDA-MB-231 and SUM1315 cells, the viral replication is strongly inhibited [53]. Similarly, we reported previously only limited tumor growth and even absence of tumor in mice xenografted with HCMV-infected HepG2 cells compared with unchecked tumor growth in mock-treated mice [54]. Inhibition of tumor growth by HCMV resulted from restricted STAT3 activation and specific activation of the intrinsic apoptotic pathway [54,55]. Recently, clearance of well-established tumors in a mouse melanoma model was obtained after injection of CMV into the growing tumor [56]. Interestingly, the development of a liver lymphoma is controlled by distant murine CMV infection [57]. Similarly, apoptosis is detected in lung tissues of xenografted mice injected subcutaneoulsy with HCMV-infected HepG2 cells [54]. These results indicate that apoptosis induction occurs both at the site of HCMV infection and/or injection and in distant organs.

A reduced relapse rate occurs in patients who reactivate HCMV early after allogeneic stem cell transplantation as treatment for acute myeloid leukemia and non-Hodgkin lymphoma [58,59,60]. The observed HCMV-induced immune modulating effects could result from increased activation of natural killer (NK) cells and CD8+ T cells, but also from HCMV-induced apoptosis of cancerous cells [61]. Interestingly, HCMV genome is undetectable in tissues (tumor, liver and lung) of mice xenografted with HCMV-infected HepG2 cells several weeks post-infection [54], and a previous study reports undetectable CMV levels a few weeks post-infection in another murine model [62]. Similar to the stalled HCMV replication cycle reported in infected MDA-MB-231 and SUM1315 cells [53], infection of HepG2 cells with HCMV results in restricted viral growth [11,12,17]. Thus, HCMV cannot infect productively cancer cell lines in agreement with multiple restrictions to HCMV replication in cells expressing oncogenic alleles [63].

Altogether, the data presented above indicate that the oncomodulation paradigm cannot always apply to HCMV infection. Besides HCMV-induced oncomodulation, in several cases, the cytotoxic effect of HCMV on the tumor growth and/or expansion has been reported. Although oncomodulation by HCMV in tumor tissues has been extensively studied, the appearance of HCMV-transformed cells in culture which induce tumor formation in vivo could indicate that HCMV belongs to the group of human oncoviruses.

## 3. Oncogenesis by HCMV

### 3.1. Human Oncoviruses

Among the 15–20% of human cancers caused by infections, several viruses have been named as human oncoviruses, including Epstein–Barr virus (EBV), hepatitis B virus (HBV), human T-lymphotropic virus-1 (HTLV-1), human papillomavirus (HPV), hepatitis C virus (HCV), Kaposi’s sarcoma associated herpesvirus (KSHV or HHV8), and Merkel cell polyomavirus [64]. Among the seven human oncoviruses described so far, five are DNA oncoviruses and share some biological features. First, the tumor suppressor proteins p53 and Rb are typically inactivated by DNA oncoviruses [65]. Second, by inactivating p53 function and Rb DNA oncoviruses overpass the G1/S check point and force the cell to enter into the S phase, which results in unregulated cell division and ultimately in tumor formation. Third, the viral integration, and to a lesser extent the viral episomes, characterize the cellular transformation by DNA oncoviruses. Although HCMV is a DNA virus, so far its role as a human oncovirus has not yet been demonstrated. We describe below several cellular and viral features that could define HCMV beyond oncomodulation close to the biological features of oncoviruses.

### 3.2. HCMV Expresses Viral Products with Potential Transforming Capacities

**Figure 2:**
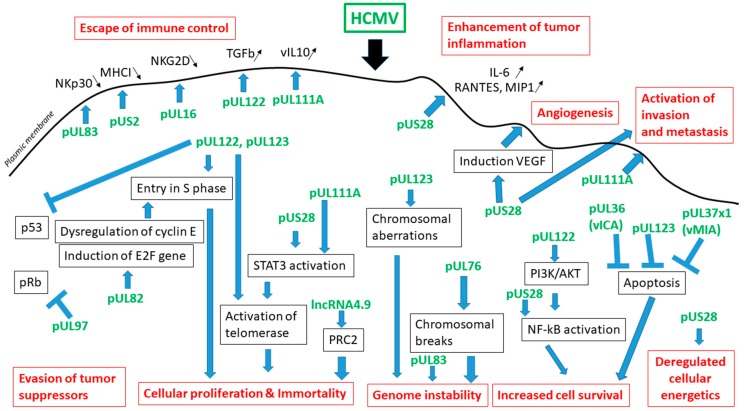
Molecular pro-oncogenic pathways activated by HCMV products. HCMV proteins are in green, cellular effectors in black, and the hallmarks of cancer in red. Black small arrows describe up- or down-regulation of cytokines and cellular proteins.VEGF-Vascular endothelial growth factor; STAT3-Signal transducer and activator of transcription 3; PRC-Polycomb Repressive Complex.

Transformation of NIH3T3 cells has been reported following stable expression of US28 gene and tumor growth occurred in mice injected with US28-expressing NIH3T3 cells [66]. Activation of IL6–JAK–STAT3 axis by pUS28 could be one of the mechanisms involved in tumor development [67]. Similarly, in primary human hepatocytes and HepG2 cells the IL-6/STAT3 axis is also activated upon HCMV infection and could favor sustained cellular transformation [12]. Both pUS28 and phosphorylated STAT3 are detected in glioblastoma tumors [67,68] (Table 1, Figure 2).

The entry into S phase is stimulated by the immediate early proteins pUL123 and pUL122 [69,70]. The proliferation of pUL123-expressing glioblastoma cells depends on p53 and Rb inhibition and PI3K/AKT activation [71]. Both in CD133+ CSC from glioblastoma multiforme and in breast tumor tissue the pUL123 protein was detected [9,47,72] (Table 1, Figure 2).

The viral cytokine cmvIL-10 is encoded by the UL111A gene, is secreted from infected cells and binds to the cellular IL-10 receptor like the natural IL-10 ligand [73]. Activation of STAT3 results from the binding of cmvIL10 to the IL-10 receptor [74,75,76,77], and has been described in breast cancer, ovarian cancer with poor prognosis, and to increase the spread of glioma cancer stem cells in malignant glioma [78,79,80]. In addition, exposure of MDA-MB-231 and MCF-7 cells to cmvIL-10 favors their proliferation, their migration and the metastatic spread due to cell surface expression of IL-10R [81,82,83] (Table 1, Figure 2).

Chromosomal breaks are present in HCMV-infected primary human foreskin fibroblasts (HFF) [84]. In addition, chromosomal breaks are induced by UL76 gene stable expression in human glioblastoma cells [85]. Parallel to chromosomal breaks, the DNA damage repair (DDR) is induced in cells infected with HCMV, as well as with other herpesviruses [86]. In agreement with such a scenario, we observed the upregulation of the expression of the ataxia telangiectasia mutated (ATM) and human MutL homolog (MLH1) genes, both involved in DNA reparation, in human mammary epithelials cells (HMECs) infected with HCMV-DB [87]. Recently, the detection of the HCMV long non-coding RNA4.9 (lncRNA4.9) in HCMV-transformed HMECs, namely CTH cells, could indicate that beyond a viral signature the lncRNA4.9 could directly participate to the transformation of epithelial cells infected with HCMV [15] (Table 1, Figure 2).

### 3.3. HCMV Fullfills the Criteria of the Hallmarks of Cancer

Recently, the hallmarks of cancer have been updated by Hanahan and Weinberg to describe the essential alterations in cell physiology that lead to cancer especially following infection with human oncoviruses [88]. HCMV infection fulfills the requirements of the hallmarks of cancer such as sustaining proliferative signals, evading growth suppressors, activating invasion and metastasis, enabling replicative immortality, inducing angiogenesis, resisting cell death, deregulating cellular energetics, avoiding immune destruction, genome instability and mutation, and tumor promoting inflammation [88]. pUL123, pUL122, and pUL97 allow the evasion of tumor suppressors p53 and pRb [15,89]. pUL122, pUL82, and pUL97 favor a sustained proliferative signal [69,90,91,92,93]. The pUL123 protein favors immortality as measured by activation of telomerase [15,94]. pUL123, pUL83, and pUL82 favor genome instability and mutation [85,95,96,97,98]. The resistance to cell death results from the expression of pUL123, pUL122, vMIA (viral mitochondria-localized inhibitor of apoptosis also known as pUL37x1), and vICA (viral inhibitor of caspase-8 activation, pUL36) [6,99,100,101]. HCMV infection deregulates cellular energetics, changes glucose and glutamine utilization, and induces the Warburg effect [102]. Several viral proteins including HCMV vIL-10 (pUL111A), pUS2, pUL16, pUL83, and pUL122 allow HCMV to avoid immune clearance [103,104,105,106,107,108,109]. HCMV infection and especially pUS28 enhance tumor inflammation, for example, by induced production of IL-6, RANTES, MCP-1, and fraktaline [66,110]. Angiogenesis is induced by pUL123 and pUS28 [111,112]. The pUS28 protein activates invasion and metastasis [113,114]. Altogether, several HCMV proteins can potentially participate to cellular transformation in a context of genomic instability and favor the spread of the tumor.

### 3.4. HCMV Triggers Pro-Oncogenic Pathways in Infected Primary Cells

HCMV has been reported by several groups to trigger pro-oncogenic pathways in the infected cells. Although upregulation of p53 has been reported in fibroblasts, hepatocytes, and HMECs following HCMV infection [12,15,115,116], the pUL122 binding to p53 in HCMV-infected fibroblasts and HMECs decreased p53 binding to DNA with inhibition of p53 activity and increased cell cycle progression and unchecked cell division [15,117]. Elevated levels of phosphoRb are observed in HCMV-infected fibroblasts and HMECs [15,118]. HCMV pUL97 phosphorylates and inactivates proteins of the Rb family and favors cell cycle promotion [118]. The pUL82 protein downregulates the Rb family proteins [91]. In HCMV-infected HMECs, the decreased detection of the Rb protein is observed parallel to the enhanced presence of the UL82 transcript [15].

One of the main characteristics of transformed cells is the sustained cell growth without cellular senescence, which depends on enhanced telomerase activity [119]. The enhancement of telomerase activity is observed in fibroblasts and HMECs infected with HCMV and leads to cell immortalization [15,94]. Increased telomerase activity could be explained by STAT3 activation observed in HCMV-infected cells [15,120].

The up-regulation of c-Myc, Akt activation, STAT3 activation, and enhanced cyclin-D1 expression are reported in several cell types following HCMV infection [21,67,68,121,122,123]. Previous reports indicate that HCMV upregulates c-Myc, c-Fos, and c-Jun in human embryo lung cells [121,122], as well as in macrophages and HMECs infected with HCMV [11,15,87]. Besides the upregulation of gene expression of the oncogenes Myc (MYC), Fos (FOS), Jun (JUN), KRas (KRAS), HRas (HRAS), and NRas (NRAS), the upregulation of transcripts of numerous other oncogenes (KITLG, MCL1, MET, MYB, NFKBIA, PIK3CA, PML, PRKCA, RAF1, RARA, ROS1, RET, ABL1, ETS1, RUNX1, RUNX3) occurs in HCMV-infected HMECs compared with uninfected cells [87]. In hepatocytes and HMECs infected with HCMV, the IL6/JAK/cyclin D1 pathway is activated with enhanced cell proliferation and upregulated transcripts of proliferation marker genes such as the Ki67 antigen gene (MKI67) and the topoisomerase 2 gene (TOPO2A) [12,15,87].

The Akt pathway is activated in HMECs infected with HCMV [15] and NF–kB activation is observed in HCMV-infected macrophages [11]. In agreement with these findings, the expression of pro-survival genes (NFKB1, REL, AKT1, PIK3C2A, BCL-2) is increased in HCMV-DB infected HMECs compared with mock-infected cells, indicating a prosurvival signal in infected cells [87]. In hepatocytes infected with HCMV, the expression of survivin is upregulated [12].

### 3.5. HCMV Transforms Epithelial Cells In Vitro and Leads to Tumorigenicity In Vivo

The most efficient assay to determine the malignancy of cells in vitro is the soft agar assay, a well-established method to measure anchorage-independent growth, the ability of transformed cells to growth independently of a solid surface, a hallmark of carcinogenesis [124]. Although several groups reported the activation of pro-oncogenic pathways in cells infected with HCMV, only limited reports describe the appearance of colonies in soft agar seeded with HCMV-infected cells. The appearance of colonies in soft agar seeded with primary human hepatocytes infected with HCMV has been reported [12]. In addition, our group observed following the infection of HMECs with the HCMV-DB strain the appearance of colonies in soft agar [15]. Interestingly, following treatment of infected cells with UV and ganciclovir, no colonies were observed [15], indicating that the soft agar colony formation requires efficient viral replication, even during a limited period of time.

**Figure 3:**
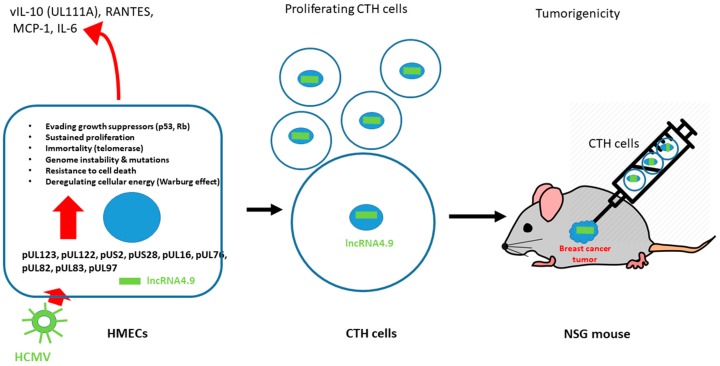
A model for HCMV oncogenesis: from viral proteins activating oncogenic pathways in infected HMECs to tumor growth in xenografted NOD/SCID Gamma (NSG) mice. CTH—CMV-transformed HMEC.

Although the appearance of colonies in soft agar seeded with HCMV-infected cells indicates cellular transformation, the tumor growth in xenografted mice following injection of HCMV-infected cells is required to directly assess the tumorigenic potential of HCMV. Recently, the injection of CMV-transformed HMECs (CTH cells), which were obtained after prolonged culture of HMECs infected with a clinical strain HCMV-DB in vitro, resulted in the development of tumors following their injection in NOD/SCID Gamma (NSG) mice [15]. The tumor growth following injection of CTH cells in NSG mice was fast [15]. In tumor biopies, a limited part of viral DNA was detected, namely the lncRNA.9 gene [15]. The HCMV lncRNA4.9 gene is also detected in biopsies of patients with breast cancer [15,125]. Thus, the HCMV-DB-infected HMEC model points toward a direct role for HMCV in oncogenesis, from viral proteins activating oncogenic pathways in infected cells to tumor growth in NSG mice xenografted with CTH cells (Figure 3).

### 3.6. HCMV Modifies the Tumorous Environment to Favor Tumor Formation

Besides a direct pro-oncogenic role of HCMV in tumor formation, the tumorous environment can be modulated by HCMV. Thus, tumor-associated macrophages (TAM), macrophages present within the tumorous environment, are present in several cancers where HCMV has been detected including breast cancer, prostate cancer, colon cancer, and glioblastoma [126]. The TAM display a M2 phenotype and produce mostly cytokines such as IL-10 and TGF-beta, which favor immune evasion and, to a lesser extent, pro-inflammatory cytokines (reviewed in the work of [127]). TAM with a M2 phenotype favors the establishment of a Th2 response, which promotes angiogenesis, tissue remodeling, and repair [126]. High TAM density is a hallmark of poor prognosis in breast cancer, colorectal cancer, and glioblastoma [128,129]. Within glioblastoma multiform, macrophages/microglia and glioma cancer stem cells can be infected with HCMV. Interestingly, only a limited copy number of the HCMV genome is detected in glioblastoma multiforme biopsies [130], indicating that non-viral biological effects may account for glioblastoma multiforme growth. HCMV vIL-10 is secreted by infected glioma cancer stem cells and favors the appearance of the M2 TAM phenotype. In addition, angiogenic factors such as VEGF, immunosuppressive cytokines such as TGF-beta, and enhanced migration of glioma cancer stem cells occur as a consequence of exposure of monocytes/macrophages to HCMV vIL10 [79]. Similarly, IL-10 and TGF-beta favor tumor migration and invasion in breast cancer and lung adenocarcinoma [131,132].

Among cell types present in breast tissue, HCMV can infect HMECs, macrophages, and fibroblasts [11,15,133]. We observed that the clinical isolate HCMV-DB displays tropism for both macrophage and HMECs [11,15]. In macrophage, HCMV-DB triggers an M2 activation state with enhanced upregulation of the proto-oncogene Bcl-3 parallel to limited viral growth [11]. Similarly, in HCMV-DB-infected HMECs, the viral replication is contained [15]. Thus, in HMECs and nearby tissue macrophages, a restricted viral replication could account for a specific tumor microenvironment, which might shape the viral fitness [134]. Further, the tumoral microenvironment could be modulated by HCMV-infected HMECs with both up- and downregulation of genes involved in angiogenesis (upregulation: IL-6, SERPINE1, THBS1, S100A4, EGF; downregulation: ID1, SLIT2) and proteolysis (upregulation: MMP9; downregulation: CST6, CTSD) [83]. In addition, the presence of tumor cancer stem cells (CSCs) has been reported to favor the propagation and invasiveness of the tumor [135]. The infection of HMECs with HCMV-DB triggers the appearance of mammospheres in culture [87] and indicates that some HCMV strains could indeed induce CSCs expansion in breast tissue in vivo. In agreement with sustained STAT3 activation observed in breast cancer [78], unchecked cell division, resistance to cell apoptosis, as well as tumor growth in mice result from IL-6/STAT3 activation in mammary CSCs [37,136]. Although some reports indicate that HCMV infection and/or viral proteins modulate STAT3 intracellular localization, IL-6 signaling, and NF-kB activation [137,138], altogether, HCMV participates to shape the tumorous environment and thereby could favor the development and spread of the tumor.

## 4. Conclusions

Although the paradigm of oncomodulation can be applied to some of the tumors infected with HCMV, oncomodulation cannot account for all the biological observations made in HCMV-infected tumors. The pro-oncogenic potential of HCMV proteins per se, the activation of pro-oncogenic pathways in HCMV-infected cells, the transformation of HCMV-infected cells in vitro, the sustained growth of CTH cells with a HCMV signature, the tumorigenicity of CTH cells injected in NSG mice, and the fulfillment of the requirement of the hallmarks of cancer all point toward the inclusion of HCMV in the list of human oncoviruses [60,139].

## Figures and Tables

**Table 1:** Human cytomegalovirus (HCMV) products with oncogenic properties.

HCMV Protein	Biological Effect	Oncogenic Feature
pUL123 (IE1)	Entry into S phase Suppression of p53 and Rb activity Dysregulation of cyclin E expression Activation of telomerase Induction of IL-1 Inhibition of apoptosis Induction of chromosomal aberrations	Cellular proliferation Evading growth suppressors Immortality Inflammation Enhanced cell survival Genome instability and mutation
pUL122 (IE2)	Entry into S phase Binding to p53 Activation of PI3K/Akt pathway Induction of TGF-beta expression	Cellular proliferation Evading growth suppressors Enhanced cell survival Increased immune suppression
pUS28	IL-6/JAK/STAT3 activation Activation of RhoA dependent mobility of U373 cells Induction of VEGF expression NF–kB activation	Cellular proliferation Tumor growth Enhanced angiogenesis Enhanced cell survival
pUL111A (vIL10)	STAT3 activation Production of homologs to immunosuppressive cytokines	Cellular proliferation, migration and metastasis Telomerase activation Increased immune suppression
pUL76	Chromosomal breaks Induction of chromosomal aberrations	Genome instability and mutation
pUL97	Phosphorylation and inactivation of pRb	Evading growth suppressors
pUL82 (pp71)	Rb downregulation Induction of E2F gene expression Increased mutation frequency	Evading growth suppressors Cellular proliferation Genomic mutation
pUS2	Inhibition of the major histocompatility complex class I expression	Escape of immune control
pUL16	Intracellular retention of NKG2D	Escape of immune control
pUL83 (pp65)	Increased mutation frequency Antagonizes the NKp30 activating receptor	Genomic mutation Escape of immune control
pUL36 (vICA)	Inhibits caspase-8 activation and apoptosis	Enhanced cell survival
pUL37x1 (vMIA)	Inhibits mitochondrial-mediated apoptosis	Enhanced cell survival
lncRNA4.9	Viral latency, binding to PRC2	Cellular proliferation and transformation
